# Deployable, Variable Stiffness, Cable Driven Robot for Minimally Invasive Surgery

**DOI:** 10.3389/frobt.2019.00141

**Published:** 2020-01-10

**Authors:** Mark Runciman, James Avery, Ming Zhao, Ara Darzi, George P. Mylonas

**Affiliations:** ^1^Human-Centered Automation, Robotics and Monitoring in Surgery (HARMS) Lab, Department of Surgery and Cancer, The Hamlyn Center, Imperial College London, London, United Kingdom; ^2^Department of Surgery and Cancer, The Hamlyn Center, Imperial College London, London, United Kingdom

**Keywords:** soft robotics, minimally invasive surgery, rapid manufacture, deployable, variable stiffness

## Abstract

Minimally Invasive Surgery (MIS) imposes a trade-off between non-invasive access and surgical capability. Treatment of early gastric cancers over 20 mm in diameter can be achieved by performing Endoscopic Submucosal Dissection (ESD) with a flexible endoscope; however, this procedure is technically challenging, suffers from extended operation times and requires extensive training. To facilitate the ESD procedure, we have created a deployable cable driven robot that increases the surgical capabilities of the flexible endoscope while attempting to minimize the impact on the access that they offer. Using a low-profile inflatable support structure in the shape of a hollow hexagonal prism, our robot can fold around the flexible endoscope and, when the target site has been reached, achieve a 73.16% increase in volume and increase its radial stiffness. A sheath around the variable stiffness structure delivers a series of force transmission cables that connect to two independent tubular end-effectors through which standard flexible endoscopic instruments can pass and be anchored. Using a simple control scheme based on the length of each cable, the pose of the two instruments can be controlled by haptic controllers in each hand of the user. The forces exerted by a single instrument were measured, and a maximum magnitude of 8.29 N observed along a single axis. The working channels and tip control of the flexible endoscope remain in use in conjunction with our robot and were used during a procedure imitating the demands of ESD was successfully carried out by a novice user. Not only does this robot facilitate difficult surgical techniques, but it can be easily customized and rapidly produced at low cost due to a programmatic design approach.

## Introduction

Endoscopic Submucosal Dissection (ESD) is a novel surgical technique for removal of large early gastric cancers with no risk of lymph node metastasis (Kang et al., 2011). ESD enables *en bloc* (in a single piece) and histologically complete resection of cancers over 20 mm in diameter, which would be removed in separate parts, known as *piecemeal removal*, if Endoscopic Mucosal Resection (EMR) were used. Piecemeal EMR (p-EMR) results in higher recurrence rates than for *en bloc* removal using ESD (Heitman and Bourke, 2017). However, the ESD procedure performed with standard endoscopes suffers from long operation times and higher complication risks in comparison with EMR (Oka et al., 2006). These disadvantages arise because of the technical difficulty associated with ESD, which is evidenced by the high number of supervised procedures that are necessary to learn the technique (Najib Azmi et al., 2016). If ESD can be achieved faster and more reliably then it may be a powerful treatment for gastric cancer, which was the second most common cause of cancer deaths in the UK in 2015 with 41,700 new cases of bowel cancer occurring annually between 2013 and 2015, being the fourth most common cancer in the UK in 2015 (Cancer Research UK, 2019).

Extending the capabilities of the flexible endoscope, the device currently used to perform ESD, may increase the uptake and accessibility of the procedure (Fukuzawa and Gotoda, 2012). Endoscopes are optical instruments that enable visualization of the inside of the body and have a variety of embodiments. In colorectal applications the endoscope used consists of a 1.5–2 m long flexible tube of 6–15 mm diameter. Depending on the model, the tip of the flexible endoscope can often be controlled using dials on the handle that use a cable system. 1–2 working channels of around 3 mm diameter that occupy a proportion of the endoscope's cross section and pass along its length are often available to deliver air, water, or small surgical instruments. Currently, ESD procedures use these working channels and the manual control of the tip to manipulate the instruments, resulting in high technical difficulty.

Standard ESD with a single port endoscope is performed in several steps: marking points surrounding the tumor, injection of solution into the submucosa below the tumor, circumferential incision in the mucosa through the marked points and dissection of the submucosal layer (Kume, 2009). An electrocautery tool is used for marking, incision, and dissection to prevent bleeding. An example of a robotic device designed specifically for ESD is the R-Scope, made by Olympus. However, in a study involving both *in vivo* porcine models and 10 human patients the procedure was still considered challenging and time-consuming, with a risk of perforating the colon (Neuhaus et al., 2006). Olympus later developed the EndoSAMURAI that featured bimanual control of two instruments, each with five Degrees Of Freedom (DOFs), which enabled suturing and shorter procedure times in comparison with a conventional endoscope (Spaun et al., 2009). The EndoSAMURAI lacked sufficient force exertion to perform more demanding retraction maneuvers and difficulty was encountered during explorative tasks (Fuchs and Breithaupt, 2012). More recently, the second generation STRAS system, originally based on the Anubiscope made by Karl Storz, was used to carry out ESD on *in vivo* porcine models and was shown to increase dissection speed compared to the manual Anubiscope (Zorn et al., 2018). Despite this, its force generation capabilities remain low, with 0.9 N bending force at the tip being measured. A review of ESD devices details a number of robotic devices as well as several non-robotic options to facilitate dissection, such as clip and line where force is exerted on the lesion to be dissected by a cable clipped to the surroundings (Saito et al., 2017). One major problem with the clip and line method is that the direction of traction is determined by the approach to the target and the patient's anatomy, because the line can only provide tension and follows the path taken. A study into the efficacy of ESD in the stomach both with the clip and line method and without it showed that perforation risk was lower due to better visualization of the tissue being dissected (Yoshida et al., 2018). However, in the overall patient population, procedure times and outcomes were not improved by using the clip and line method. ESD is still being carried out with single channel flexible endoscopes as current approaches, either robotic or non-robotic, have not provided sufficient improvement.

This article describes the rapid design and manufacture of inflatable, deployable, variable-stiffness structures that support the cable-driven bimanual robotic platform known as CYCLOPS, designed to perform advanced surgical techniques such as ESD through a single port or a natural orifice (Mylonas et al., 2014). The CYCLOPS concept allows for ESD to be carried out without the need for extensive training because of the intuitive, two-handed control of surgical instruments (Vrielink et al., 2018). The assembly is designed as an attachment to a standard flexible endoscope, leaving its working channels free and adding two robotically controlled instruments.

The use of an inflatable structure to support the CYCLOPS parallel robotic platform means that large changes in volume can be achieved, so the endoscope can remain relatively unencumbered until the CYCLOPS system is to be deployed and used. The entire undeployed assembly can be wrapped around a flexible endoscope and pass through a standard trans-anal port, or alternatively could be pulled to the end of the endoscope along the outside by a cable passing through a working channel.

Rigid, bulky, and sharp instruments can cause unwanted damage to soft tissue in the restricted space of the lower gastrointestinal tract, but many instruments and robotic devices used in minimally invasive surgery are made from rigid materials. Conventional robotic mechanisms can achieve high precision, accuracy and predictability because they employ rigid materials throughout their structure. Soft robotics relies on materials with low stiffness relative to the environment in which they will be used but, in many cases, this means materials used in a robot's construction have a non-linear strain profile (Polygerinos et al., 2017). Non-linear strain profiles are undesirable in robots because they can make the end-effector uncontrollable (Lipson, 2014). For a robotic device to be used in MIS, the benefits of both soft and rigid robotic systems are desirable. A soft device would reduce the risk of damaging soft tissue but, in contrast, a rigid device can exert higher forces and achieve predictable, precise motion.

The majority of soft robotic devices designed for surgery are fluid actuated, elastomer, continuum style robots, and the fluid medium used most commonly is air (Runciman et al., 2019). The STIFF-FLOP project was one of the first that demonstrated the use of soft robotics in minimally invasive surgery and has inspired a great deal of research. Despite their intrinsic safety benefits, common limitations of soft devices include low force exertion, poor controllability, a lack of integrated sensors and complex manufacture (Rus and Tolley, 2015). A large amount of research is focused on overcoming these problems and controlling a device's stiffness is an important part of this.

Methods to vary the stiffness of flexible medical devices include pneumatic fluid actuation, hydraulic fluid actuation, granular jamming, laminar jamming, antagonistic combinations of cables and pneumatic chambers, and phase transitioning materials (Zhao et al., 2016). Both pneumatic actuation and granular jamming were used in a cadaver trial to provide visualization during Total Mesorectal Excision (TME) using a two-module STIFF-FLOP robot (Arezzo et al., 2017) and pneumatic actuation alone was used to actuate and stiffen a similar two module STIFF-FLOP robot in a separate cadaver study of the same procedure. Hydraulic actuation was used in a different iteration of the STIFF-FLOP robot that permitted force estimation on the tip (Lindenroth et al., 2017). Laminar jamming has been used to rigidify a tubular manipulator that was designed to support other continuum or serpentine robots for MIS (Kim et al., 2013). Granular and laminar jamming can increase the cross-sectional area of a device in its non-rigidified state compared to fluidic actuation alone and adding multiple actuation methods increases the design complexity. However, granular jamming and additional actuation methods can produce greater changes in stiffness than fluid actuation (Manti et al., 2016). Both cable and pneumatic actuation were used antagonistically in another STIFF-FLOP design to increase stiffness, achieving improved results compared to granular jamming (Shiva et al., 2016). Low Melting Point Alloys (LMPAs) were used in an overtube for endoscope devices and can transition from a rigid to flexible state in around 18 s (Zhao et al., 2016). Limitations of LMPAs included the toxicity of the materials, and the bulk added by the thermal insulation that protected the surroundings from elevated inner temperatures.

Soft robots constructed from elastomers often require complex mold assemblies and have lower packing efficiencies than our foldable designs because the chambers incorporated within them occupy larger volumes when unpressurised. Less space is available for dedicated channels for therapeutic and/or diagnostic instrument delivery if the actuation or stiffening mechanisms take up a large proportion of the cross-sectional area. The ability to fit through a 12–15 mm diameter opening will allow a device to fit through trocar port for use in laparoscopic applications (Jiang et al., 2013).

Using a laser welding manufacturing technique, we have the capability to rapidly and economically produce low-profile inflatable structures by heat-sealing thermoplastic sheet material in specific weld patterns. This means devices can have a smaller size when not in use. Additionally, this manufacturing method has very low consumables cost, it is reliable and typical welding steps for designs in this article can be completed within ~3 min. This method makes single use, disposable soft robotic devices viable.

Designs for deployable structures, in the form of inflatable hollow regular prisms of varying side number, side length, total length, and diameter, can be generated using our programmatic design approach. The outer sheath can also be rapidly customized based on the structure it is to fit around. By choosing the parameters for the structure and the entry point positions, new deployable CYCLOPS designs can be rapidly generated.

The following sections detail the programmatic design, laser welding manufacture, CYCLOPS robot construction, testing methods, and results. In the results section, the variable stiffness properties, force exertion capabilities and pre-clinical testing are described.

## Materials and Methods

**Figure 1:**
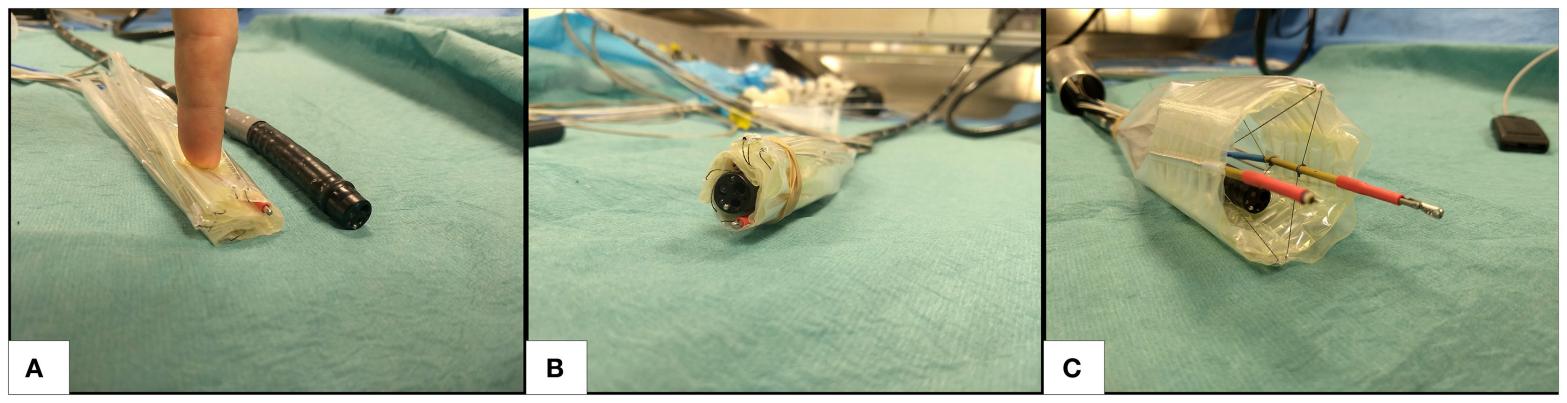
Deployable cable-driven parallel robot **(A)** folded beside 12 mm diameter flexible endoscope (black) **(B)** undeployed robot placed around flexible endoscope **(C)** inflatable structure deployed and force-transmission cables pretensioned to allow the two surgical instruments (anchored inside gold and red shafts) to be controlled by changing each cable length.

This section describes the basic principle of how the instruments within the CYCLOPS are manipulated and some of the design considerations of the device. Figure 1A shows our robot in a folded and undeployed state beside the tip of a 12 mm diameter flexible endoscope, then wrapped around the tip in Figure 1B as it might be used during navigation, and Figure 1C displays the deployed robot with two instruments ready for use. Our inflatable CYCLOPS robot provides control of these surgical instruments, which allows for a novice user to perform tasks similar to those performed during ESD. Motion of each instrument is achieved by varying the lengths of several force-transmission cables, which are attached at specific points to tubular end-effectors that carry the instruments. Six cables are used for each of the left- and right-hand instruments, which constitute a pair of parallel manipulators that occupy a single deployable, inflatable support structure.

There are two main elements to this design: the inflatable structure and the cable sheath around it. The hollow hexagonal prism shaped structure can increase its volume and stiffness to deploy and resist the forces exerted on it by the cables, while the cable sheath determines the points that the cables enter the interior of the structure. These are both manufactured using a laser welding system that heat seals thermoplastic sheets and, therefore, allows airtight chambers to be formed. Constructing the robot from low-profile sheet materials means that the robot assembly can be folded to a small volume when uninflated. Furthermore, the cable configuration is highly flexible when the robot is not in use, a design that takes inspiration from the field of soft robotics and motivation from a need for safer medical instruments and devices.

To take advantage of the compliance of low stiffness materials and also the predictability of rigid structures, we chose to use a variable stiffness method for the core structural element. Despite the lower stiffness variation compared to other methods, fluid actuation was chosen due to its simplicity, the possible stiffness values achievable, reversibility, and the high changes in volume that can be achieved. Air was chosen as the actuation fluid because the weight change as a chamber is pressurized with air is low, as opposed to hydraulic actuation where water being pumped into a thin-walled chamber can be several times heavier than the chamber itself. A device that becomes heavier as it transitions from a non-operational to an operational state is not desirable in the context of the large intestine because the soft tissue may deform, affecting localization. By pressurizing the air in the chambers created by heat-sealing multiple thin sheets of thermoplastic material, the flexible, low-profile sheets can reversibly transform into a rigid three-dimensional structure. Heat-sealing sheet materials with rhombus shaped chamber patterns has been shown to produce fluid actuated hinges whose torque increases with input pressure up to bursting point (Ou et al., 2016) and bending motion can be produced by pressurizing inflatable beams placed at the axis of a hinge (Niiyama et al., 2014). These concepts influenced the hollow prism design used as the structure of our inflatable CYCLOPS.

To take advantage of the compliance of low stiffness materials and also the predictability of rigid structures, we chose to use a variable stiffness method for the core structural element. Despite the lower stiffness variation compared to other methods, fluid actuation was chosen due to its simplicity, the possible stiffness values achievable, reversibility, and the high changes in volume that can be achieved. Air was chosen as the actuation fluid because the weight change as a chamber is pressurized with air is low, as opposed to hydraulic actuation where water being pumped into a thin-walled chamber can be several times heavier than the chamber itself. A device that becomes heavier as it transitions from a non-operational to an operational state is not desirable in the context of the large intestine because the soft tissue may deform, affecting localization. By pressurizing the air in the chambers created by heat-sealing multiple thin sheets of thermoplastic material, the flexible, low-profile sheets can reversibly transform into a rigid three-dimensional structure. Heat-sealing sheet materials with rhombus shaped chamber patterns has been shown to produce fluid actuated hinges whose torque increases with input pressure up to bursting point (Ou et al., 2016) and bending motion can be produced by pressurizing inflatable beams placed at the axis of a hinge (Niiyama et al., 2014). These concepts influenced the hollow prism design used as the structure of our inflatable CYCLOPS.

Previous designs of the CYCLOPs robot were able to exert forces of 65 N (Mylonas et al., 2014) and 46.39 N (Vrielink et al., 2018), however, this inflatable version of the design is expected to exert lower forces. In a study into the forces required to manipulate the gastric mucosa in a porcine model a maximum force of 2.26 N was required to stretch the mucosa, with the connective tissue to the stomach left intact, and a 1.13 N estimated mean force observed (Traeger et al., 2014). A separate study showed that <1.5 N was required for tissue manipulation in Transanal Endoscopic Microsurgery (TEM) (Ranzani et al., 2015). The robot should be able to exert forces above these values to meet the requirements of use in this application.

### Programmatic Design

In our multiple layer, multiple chamber design, one layer consists of a series of inflatable beams that form the circumference of a hollow prism structure, while a second layer consists of a series of stiffening beams perpendicular to those on the first layer and situated at each corner, running along the entire length of the inner surface. The stiffening beams increase the stiffness of the structure when inflated. Maintaining a low number of air supply tubes while still delivering variable stiffness capabilities was important for the application of these devices in MIS where small size is beneficial, so this two-layered, two-chambered design was chosen.

**Figure 2:**
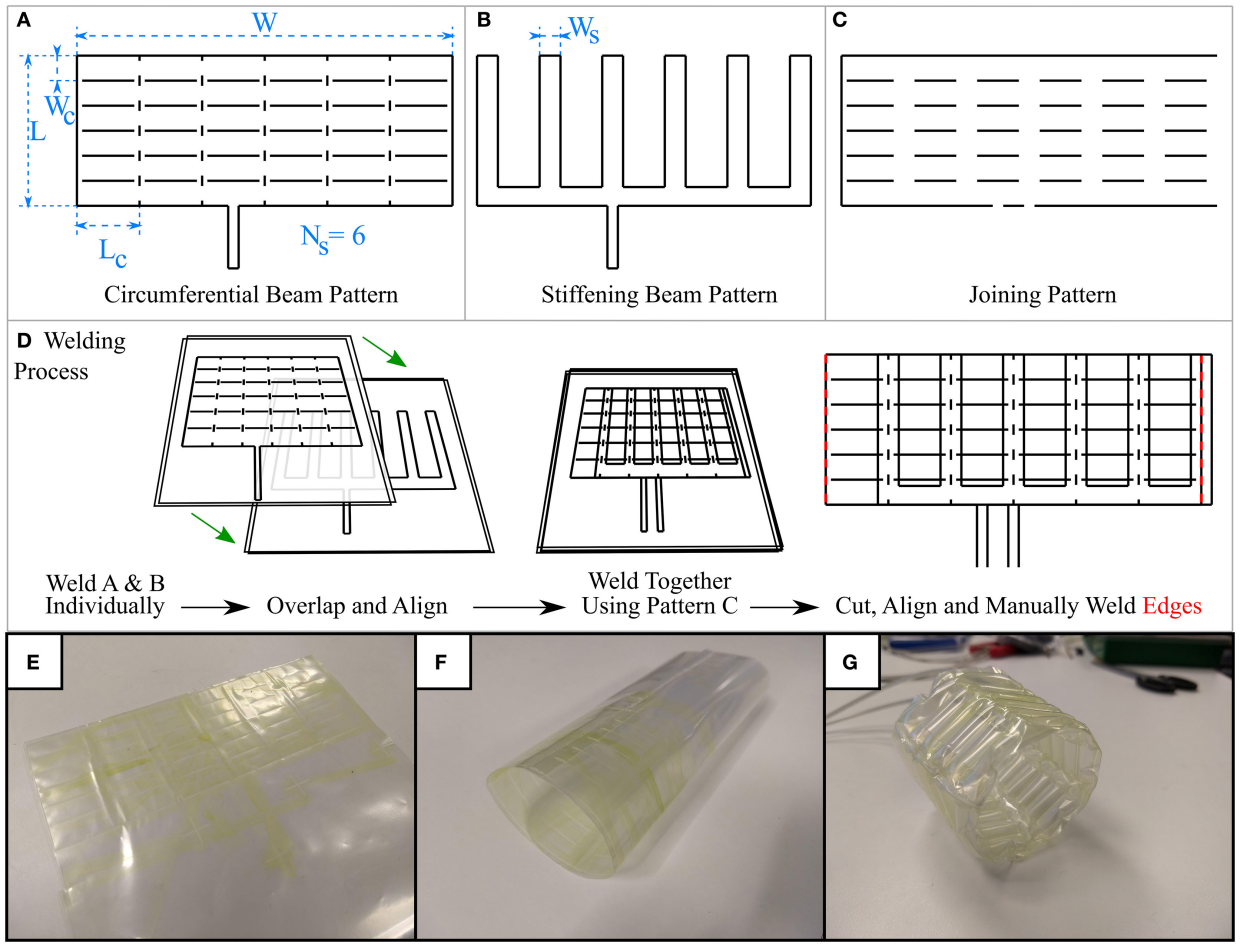
The three weld designs generated using programmatic approach **(A)** the weld pattern to produce a circumferential beam layer, with key dimensions highlighted **(B)** weld pattern to produce stiffening beams at the prism vertices, stiffening beam width highlighted **(C)** weld pattern to join layers produced in A and B where their weld patterns intersect **(D)** process of welding layers together, followed by **(E)** cutting and **(F)** manually welding along the red lines in **(D)** results in a cylindrical shape **(G)** Lastly, the completed inflatable structure can be pressurized, taking its prismatic shape.

Here, a layer will refer to the weld patterns created in a single welding process. Two or more thermoplastic sheets may be welded together to create airtight seals with a number of individual chambers, making one layer such as that in Figure 2A. In an additional step, a second layer also consisting of two or more thermoplastic sheets can be manufactured in a separate process, for example Figure 2B. To join two layers, one layer is aligned on top of the other and they are welded together at points where their weld patterns intersect or where welding would not impede an existing chamber on any layer. The joining pattern in Figure 2C achieves this when patterns A and B are aligned. Laser welding two layers simultaneously, welds all their thermoplastic sheets together due to their low thickness and the rapid heat transfer between them and is illustrated in Figure 2D, with the final result of joining two layers visible in Figure 2E. Masking individual layers to prevent laser exposure and, therefore, heat-sealing may also enable even more complex designs. The capability to produce multiple chambers on individual layers and to join multiple layers opens up a vast range of design possibilities, such as combining each layer's functionality or creating complementary bending behaviors. It should be noted that joining more than two layers is also possible.

With reference to Figure 2, three weld patterns are necessary for this design: one to produce the circumferential beams on one layer, one to produce the stiffening beams on a second layer, and one weld pattern to join these two layers where their weld patterns overlap. The parameters circumferential beam length, L_c_, circumferential beam width, W_c_, number of circumferential beams N_c_, stiffening chamber width, W_s_, number of sides, N_s_, describe some key dimensions of the weld patterns that make our variable stiffness structure design. By altering these parameters, the total length, circumference, and wall thickness of the resulting N_s_-sided inflatable regular hollow prism can be changed. We wrote a program in the Python programming language that generates the three weld patterns (circumferential beam pattern, stiffening beam pattern, and joining pattern) in DXF format based on these parameters, which can then be converted to *gcode* or *urscript* such that they can be manufactured by our laser welding system that is described in the next section. Figure 2 displays some example weld patterns, the key parameters set by the user, and an overview of the welding process.

The ability to rapidly design and manufacture new variable stiffness inflatable structures enables their customization. Using a customisable structure for a CYCLOPS robot allows the robot itself to become customisable. In the context of MIS, a robot that can be customized to the patient or the patient's condition would help to avoid unintentional harm and to reduce complications. Different ethnic groups show variation in both the length and diameter of each part of the large intestine (Khashab et al., 2009) so, in its simplest form, this could mean sizing the outer diameter of the deployable robot to the diameter of a patient's large intestine to reduce stretching or risk of perforation. A longer structure length increases the maximum possible reach of the CYCLOPS instruments, which may be appropriate if a large reach is required for a given surgery, for example an early gastric cancer over 20 mm in diameter that would otherwise be removed piecemeal.

In a systematic review of 22 separate studies covering 2,841 cases in which ESD was used, the median of the mean tumor size was 32.4 mm, with a range of 6.2–43.6 mm (Repici et al., 2012). This value can be used to influence the design of the inflatable structure to ensure the workspace of the instruments can cover an area of this diameter. Reducing the cross-section of the inflatable structure will reduce the instruments' workspace for purely translational motion. The workspace is increased when rotation is permitted, and longer instruments can further increase the workspace at the cost of accuracy of tip position control.

The inflatable structure used in the CYCLOPS robot in this article had the following parameters:

- Circumferential beam length, L_c_ = 30 mm
- Circumferential beam width, W_c_ = 10 mm
- Number of circumferential beams, N_c_ = 6
- Stiffening chamber width, W_s_ = 12 mm
- Number of sides, N_s_ = 6
- Length, L = 60 mm
- Width, W = 180 mm.

These parameters allow the inflated structure to be approximated to a hollow hexagonal prism with side length 28.5 mm, resulting in an ~24 mm purely translational horizontal range of motion for each instrument in the ideal case.

The chosen hollow prism shapes are developable i.e., they can be “unfolded” onto a planar surface, so they can be obtained by joining two ends of the outer rectangles that appear in the 2D weld patterns. We used a soldering iron to manually weld the ends of the completed multiple layer rectangular weld designs, as shown in Figure 2D, taking care not to alter previously welded chambers. Small vertical welds were added in order to induce buckling of the circumferential beams at the vertices of the prism, allowing the shape to form when inflated.

To demonstrate the adaptability of the manufacturing method and to investigate how structural stiffness changes with internal pressure and with stiffening chamber width, two deployable structures were fabricated that were identical except for the width of their stiffening chambers. One was made with stiffening chambers of width W_s_ = 12 mm and the second made with W_s_ = 15 mm. All other dimensions were kept the same and the two layers of both the circumferential structure and stiffening chamber designs were welded together in the same way.

### Laser Welding Manufacture

**Figure 3:**
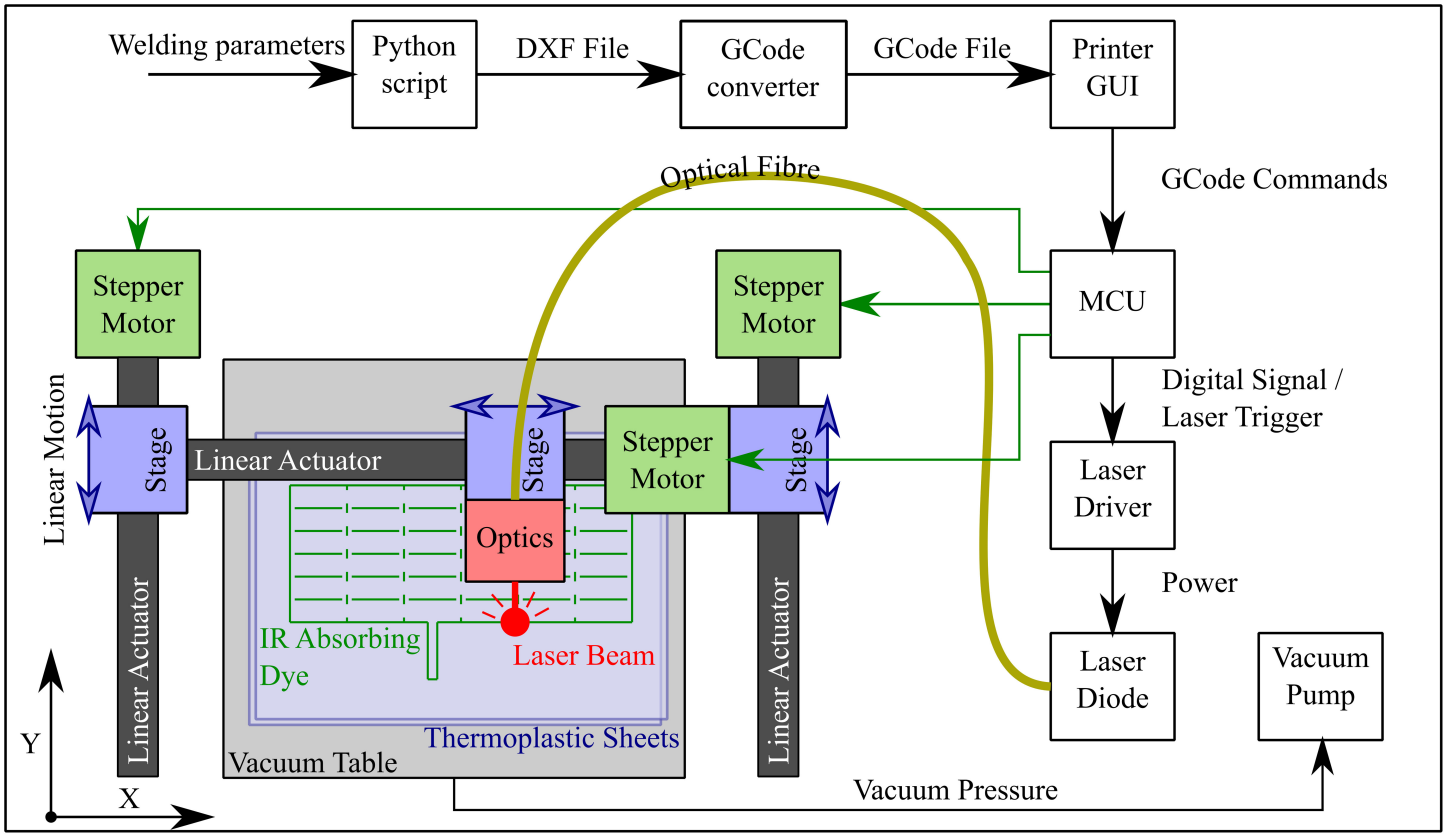
Workflow and schematic of laser welding system showing the progression from the welding parameters input by the user to *gcode* commands interpreted by the microcontroller (MCU), which controls not only the motion of the optics but also the triggering of the laser diode. The Cartesian robot constitutes three linear actuators whose stages are driven by belts and timing pulleys coupled to stepper motors.

**Figure 4:**
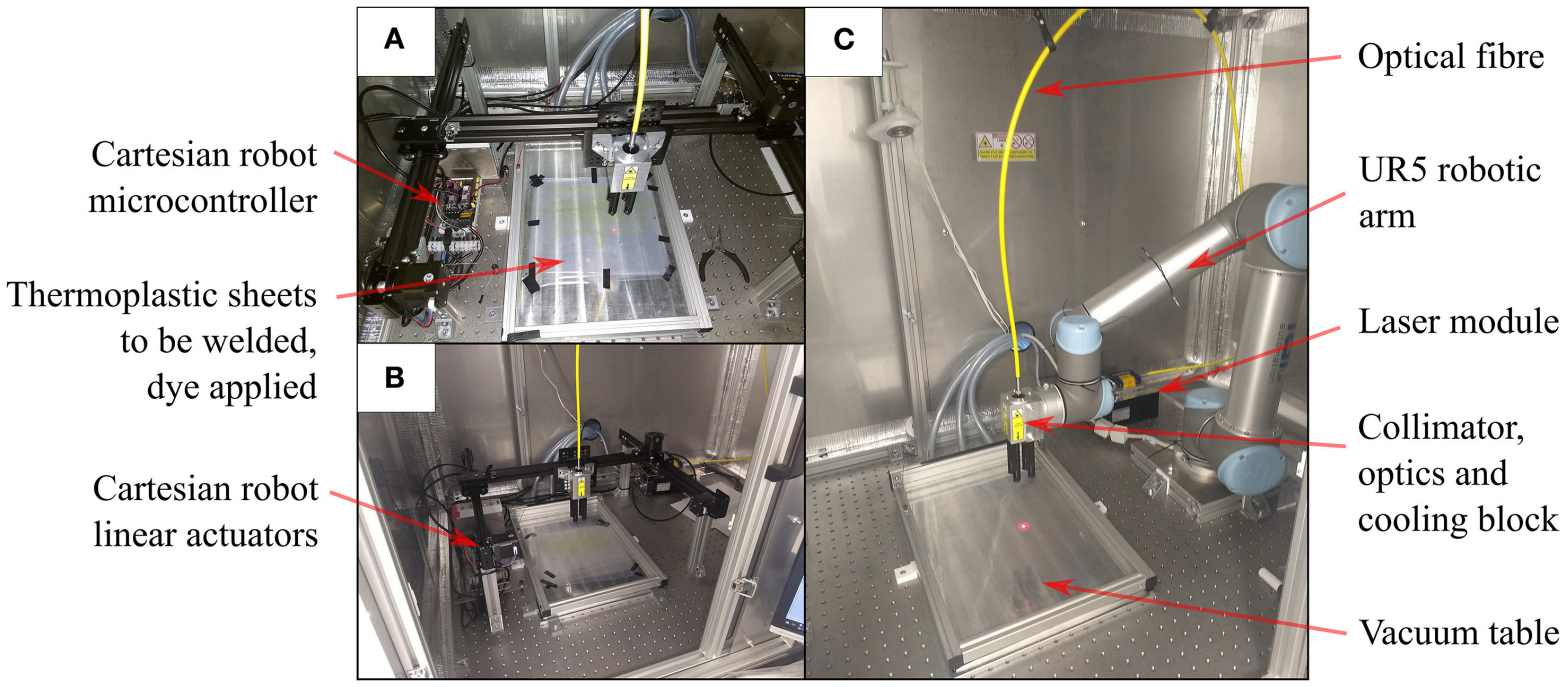
Laser welding for planar designs **(A)** Cartesian robot with red pilot laser beam tracing a weld pattern at the interface between thermoplastic sheets **(B)** Cartesian robot within safety cabinet **(C)** Six DOF robot arm setup in the same cabinet for planar welds on the vacuum table.

Taking inspiration from 3D printing technology, we constructed a laser welding system capable of selectively welding thermoplastic sheet materials together in two-dimensional weld patterns. A Cartesian robot, shown in both Figure 3 and Figures 4A,B, was built using readily available and economical parts that are controlled by a readily available microcontroller (MCU) (Arduino Mega 2560, Arduino, Italy) configured with *Marlin* firmware. The open source 3D printing software *Pronterface* is used as a Graphical User Interface (GUI) for the system. The end effector of the Cartesian robot has a workspace of 356 × 296 mm and guides a collimated laser beam that is focused to a 0.8 mm diameter spot at the surface of a vacuum table. The beam is generated by a 940 nm wavelength laser diode (LuOcean Mini LU0940D250-D, Lumics, Germany) controlled by a laser driver (LuOcean LU_DR_AD18A08VAAC, Lumics, Germany) and passes through an optical fiber to an optical assembly including the collimator and lens, which is mounted inside an Aluminum block. An infrared (IR) absorbing dye (Clearweld LD940B, Crysta-Lyn Chemical Company, USA) is applied to the upper surface of one of the thermoplastic sheets using a pen applicator attachment to the robot's end effector, which deposits the dye where welds are to be made. Additional thermoplastic sheets that cover the area where dye was applied are placed on top and an acetate sheet covering the entire area of the vacuum table is placed on top of that to create an airtight seal. The boundary of the vacuum table is also clamped to reduce airflow. The vacuum table is connected to a pump (Rubin 90, Briwatec, Germany) that produces 15 kPa of negative pressure, clamping the thermoplastic sheets together. As the focused laser beam passes over the dye during welding, the dye absorbs the laser light and heats up, rapidly and locally melting the nearby surrounding thermoplastic sheets and joining them. The weld pattern for the circumferential beam layer used to construct the CYCLOPS took 2 min 23 s to execute, the stiffening beam layer taking 1 min 26 s and the joining weld 1 min 36 s to complete. In each case the same time is taken when running the *gcode* program to apply the infrared absorbing dye before exposure to the laser.

The thermoplastic sheet used was a polyethylene (PE) polyethylene terephthalate (PET) triple laminate, PE/PET/PE, of thickness 30/60/30 μm (PE/PET/PE solvent bonded laminate, A. Warne). The welding speed was set at 23 mm/s and the laser current set at 6.5 A, corresponding to a laser power of 15 W. Continuous welds of ~1 mm track thickness were achieved with these settings. As displayed in Figure 4C, this method of laser welding has also been achieved using a 6 DOF robotic arm (UR5, Universal Robots, Denmark) (Runciman et al., 2018) but in this case the 2 DOF Cartesian robot was used to demonstrate that this manufacturing technique can be executed with lower budget equipment, permitting widespread uptake.

Laser welding avoids the wear and tear of moving parts in contact and achieves high quality, reliable, and repeatable welding results, although the thermoplastic sheets must be free of dust before welding as this can prevent an airtight seal being formed. The laser power and welding speed are controllable and easily adjustable, meaning that the same system can be adapted for new designs and use with other materials. The use of simple designs that are easily manipulated and robotic systems to guide the laser beam enables rapid and adaptable designs. Expensive, bespoke heat press stencils, or dies are also avoided.

#### Pneumatic Control

Two syringe pumps were constructed to be able to individually control the pressure within separate chambers. A high-resolution pressure sensor (MS580314BA01-00, TE Connectivity, Switzerland) reads the pressure within the supply tube and a microcontroller (Arduino Uno Rev 3, Arduino, Italy) controls a stepper motor that moves the syringe plunger using a bang-bang control strategy. This approach is precise enough for this application, it is quiet during operation and has a small footprint.

The stiffness of the inflatable structures is related to the internal pressure, so the pressure was controlled at a constant value to maintain the rigidity or compliance of the structure and additionally to reduce the risk of bursting.

For each of the fluid actuated devices, tubing of 3 mm outer diameter was used to connect the inflatable chambers to the pumps. To connect to the laser welded inflatables, the tubing was inserted into inlets that were part of each weld pattern design and heat sealed onto the thermoplastic sheet using a hot air gun and heat shrink sleeve. This was chosen because it resulted in fast, economical and low-profile connections.

### Construction of CYCLOPS Bimanual Robotic Device

The CYCLOPS consists of a structure, a series of cables and a set of “overtubes”. The cables attach to the overtubes, which hold instruments, and transmit forces in order to move the instruments. The cables themselves pass through 1.4 mm diameter Bowden cables (Round Wire Coil, Asahi Intecc, Japan) that are anchored at specific entry points on the structure. The structure provides support such that the Bowden cables at the entry points do not move inwards toward the overtubes. To reduce friction with the Bowden cables, PTFE tubing was first fed through them before the force transmission cables. Having already constructed a suitable deployable structure, a sheath was manufactured that would anchor the Bowden cables and maintain precise spacing between the anchor points around the structure's perimeter because this is of importance to the kinematics of the end-effectors. Overtubes of 60 mm length were made from brass tubing with 3 mm inner diameter.

Therapeutic or diagnostic instruments of diameter 3 mm can be integrated into the overtubes of the CYCLOPS. This means that standard flexible instruments that fit down the working channel of flexible endoscopes can be used. The overtubes are required because the instruments themselves would deform excessively if connected directly to the force transmission cables, making them uncontrollable.

**Figure 5:**
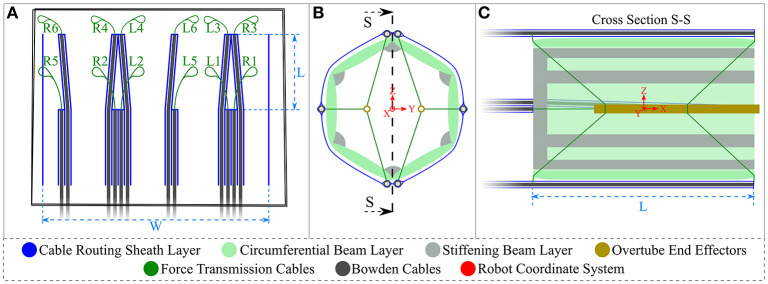
Cable sheath and CYCLOPS schematic diagram **(A)** cable sheath design in blue showing Bowden cables and force-transmission cables, where the vertical line welds on the right and left are later manually welded to produce a cylinder **(B)** front view of CYCLOPS where the inflatable structure and sheath are in place, the cables are attached to the brass overtubes and also showing the origin of the coordinate system in red **(C)** cross sectional view showing entry points of cables to the structure interior, the attachment points on the overtubes and the robot's coordinate system in red. Not to scale.

The sheath was designed as a hollow cylinder to be placed around the inflatable structure, with each Bowden cable/PTFE tubing/force transmission cable assembly running through its own designated channel. Six cables were used per overtube, so 12 channels were necessary. The vertical line welds at each side were vertically aligned and manually welded together using the same method as for the inflatable structures, resulting in a cylinder of known diameter. The Bowden cable assemblies were pushed through the 4 mm channels in their respective order, as illustrated in Figure 5, and a small hole made at the end of each channel to constrain the Bowden cable and for the force transmission cable to pass through. These holes represent the entry points. The placement of each entry point on the sheath is such that two overtubes can be connected to the force transmission cables and these cables will never collide, while the workspace of each overtube is an equal mirror image of the other. The entry point configuration delivers 5 DOF to each overtube, namely translation in the X, Y, and Z directions and rotation around the Y and Z axes. The local coordinate system of the robot is found at the center of the support structure and is depicted in Figure 5.

To fit the inflatable structure inside the sheath, the overtubes were connected to the force transmission cables at the rear attachment points with the cables passing through the rear opening of the deployable structure that had been placed inside the sheath, then the overtubes were connected to the cables at their front attachment points through the front opening of the structure. The rear cables pass through slots cut into the excess plastic at the back of the inflatable structure to prevent relative angular displacement between the sheath and the structure. The laser welding step of the sheath manufacture took 1 min and 42 s to complete. The completed, undeployed CYCLOPS robot has a mass of 24 g.

The force-transmission cables of the CYCLOPS robot were then connected to the motor unit of the control system as described in Vrielink et al. (2018), which controls the length of each cable individually to realize the desired pose, the position and orientation, of each instrument. The desired instrument poses are set using two haptic controllers (Geomagic Touch, 3D Systems, USA), and five of their six DOFs are used, i.e., the rotation around the handle of the controller is ignored because the cable configuration used does not permit rotation of the overtube around the X axis. A grasping instrument and a monopolar diathermy instrument for flexible endoscopes (VIO 200D, Erbe Elektromedizin GmbH, Germany) were fixed in place in the left and right overtubes, respectively, using heat shrink tubing. Finally, a 12 mm diameter flexible endoscope (Karl Storz) was inserted through the center of the CYCLOPS and the spare sheet material at the rear of the cable sheath was folded around and affixed to the endoscope.

### Stiffness and Force Exertion Measurement

#### Variable Stiffness Structure Stiffness Measurement

**Figure 6:**
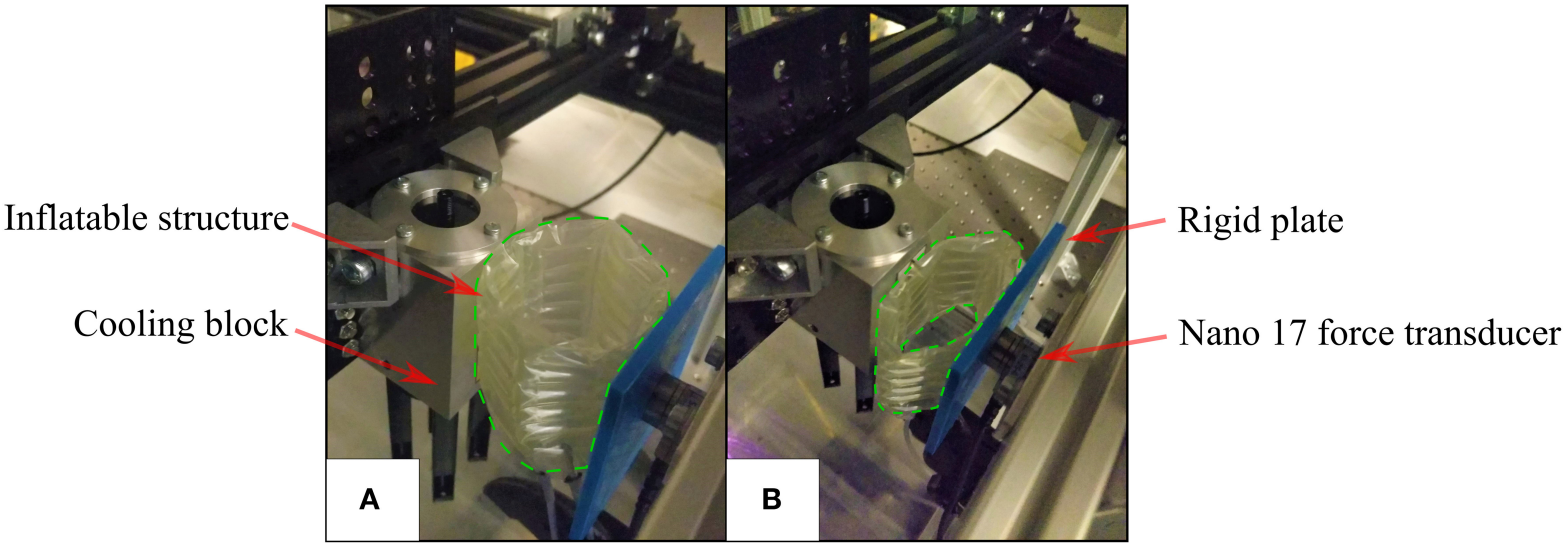
Radial stiffness measurement setup **(A)** the initial position where the Aluminum block is in contact with the inflatable structure (enclosed by green dashed line) **(B)** the inflatable structure deflecting and exerting force on the force transducer during testing.

The deployable structures were compressed between two vertical surfaces, one of which was attached to a force/torque sensor (Nano 17, ATI Automation, USA) connected to a 16-bit NI USB-6259 DAQ (National Instruments, USA), the other surface being the aluminum block attached to the end effector of the Cartesian robot, as shown in Figure 6. The aluminum block was moved into contact with the inflatable structure and a simple program was run to compress the structure by 10 mm, then compress by a further 10 mm, pause for 3 s and retract 10 mm 10 times before retracting to the start position. Before the procedure was started, the two syringe pumps were used to set and log the pressure in the inflatable chambers. The force/torque sensor was used to log data during this process, with the 3 s pause between each repetition added to the program in order to allow the force values to settle. The stiffness was then calculated using the mean of the last 10 samples of the settled resultant force value before the next cycle started.

Due to the construction of the multiple-layer, multiple-chamber scaffold, the pressure in each of the two chambers can be controlled independently. Stiffness testing was carried out when both chambers were pressurized from a single source at a range of given pressures and then repeated with the pressure in the circumferential chamber held constant at 1,500 mbar absolute while the pressure in the stiffening chamber was varied. Additional tests were carried out to investigate asymmetrical radial stiffness of the structure due to the discontinuity caused by the manual weld that seals the ends together. To test for this, the method described earlier was used and a structure with 12 mm stiffening chambers inflated with a pressure of 1,500 mbar absolute in both the circumferential and stiffening chambers. The radial stiffness was calculated when each of the six faces was in contact with the plate coupled to the force sensor.

#### Force Exertion by CYCLOPS Instruments

**Figure 7:**
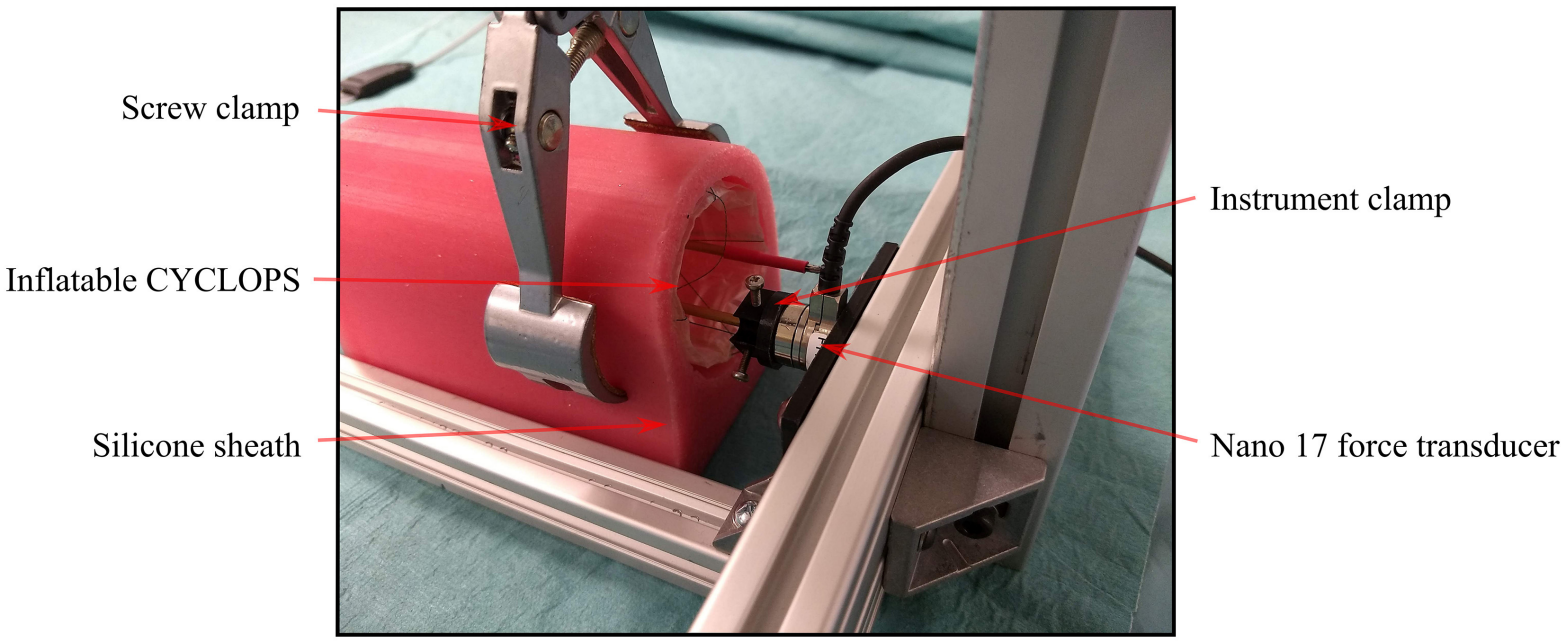
Force exertion test setup showing the right overtube rigidly clamped to the force transducer and the CYCLOPS structure held by a clamp.

The force exertion capabilities of end-effectors of the inflatable CYCLOPS robot were examined using the force/torque transducer. The right overtube with its surgical instrument temporarily removed was clamped to the surface of the force transducer, which was coupled to the structure of the CYCLOPS robot. A silicone sheath was used to constrain the inflatable structure without deforming it unevenly, which would influence the kinematic model that the control of the instruments is based on. The CYCLOPS structure was pressurized to 2,000 mbar absolute throughout each test, an image of which can be seen in Figure 7.

Once the instrument was clamped in place, the master controller was moved and the motion mapped onto the specific axis for which the force exertion capabilities were to be measured such that the instrument, if unconstrained, would have moved only in that direction without rotating. The forces were measured when the master controller was moved through its entire range of motion in each of the X, Y, and Z directions.

## Results

### Variable Stiffness

**Figure 8:**
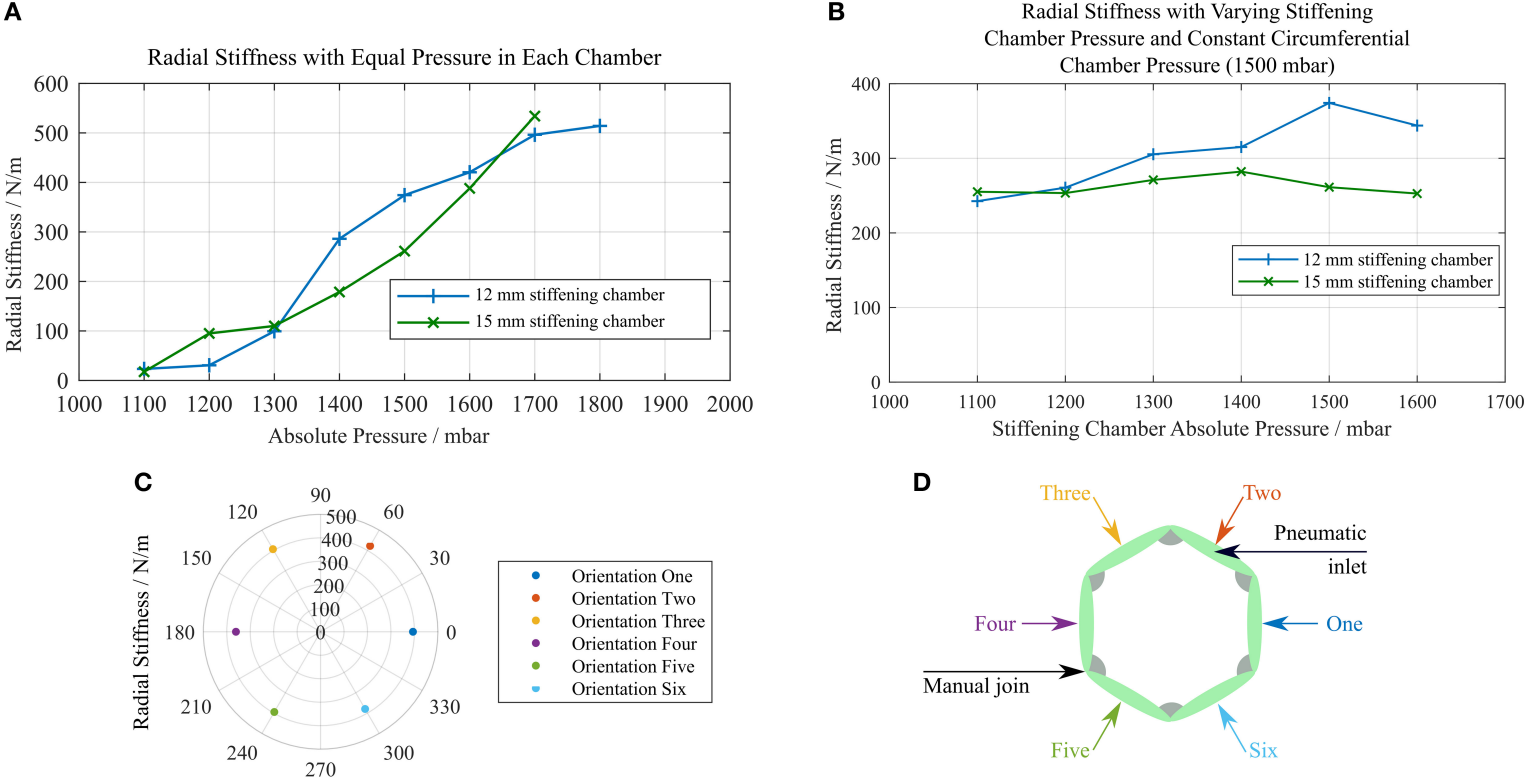
Plots of radial stiffness against chamber pressure **(A)** stiffness of identical structures with 12 and 15 mm stiffening chambers with equal pressure in each layer **(B)** stiffness of each structure with a constant circumferential chamber pressure of 1,500 mbar and varying stiffening chamber pressure **(C)** radial stiffness measured at six different orientations **(D)** diagram showing which face was in contact with the force sensor for each orientation in **(C)**.

The values of radial stiffness calculated when both chambers were at equal pressure for each of the two variable stiffness structure designs can be seen in Figure 8A. The values of radial stiffness when the circumferential beams were inflated to 1,500 mbar and the pressure in the stiffening chambers was varied can be seen in Figure 8B.

As the pressure was increased, the structures were able to restore their original shape to a greater degree. Deformations made at low pressures remained after the external force that caused them was no longer present. This can be seen in the force data collected as a decrease in the measured force over the 10 compressions of the structure. The percentage difference between the minimum and maximum radial stiffness values across all orientations of the structure was 15.69%, and there was no pair of orientations that showed a notably lower radial stiffness across an axis of symmetry as shown in Figure 8C. Figure 8D displays the face of the structure in contact with the force sensor for each orientation tested.

The burst pressures for each chamber of the 15 mm stiffening chamber structure were 2518.8 and 2572.1 mbar absolute for the stiffening chamber and circumferential beam chamber, respectively. The burst pressure is dependent on the weld pattern geometry, where larger spaces between welded regions result in failures at the welds themselves at lower pressure. In our experience with this solvent bonded PE/PET/PE triple laminate material, failure has most often been manifested as a delamination of the central PET layer from the outer PE layers, which has led to the rapid propagation of a tear. Materials with higher delamination strength will improve these results.

### Force Measurement of CYCLOPS End-Effectors

The maximum forces exerted by the end-effectors in each direction during the force exertion tests are displayed in Table 1. See Figure 5 for a depiction of the local coordinate system.

**Table 1:** Maximum forces measured in each axis during force exertion tests.

**Direction**	**+X**	**–X**	**+Y**	**–Y**	**+Z**	**–Z**
Force/N	7.62	8.29	2.76	2.93	6.23	5.53

**Figure 9:**
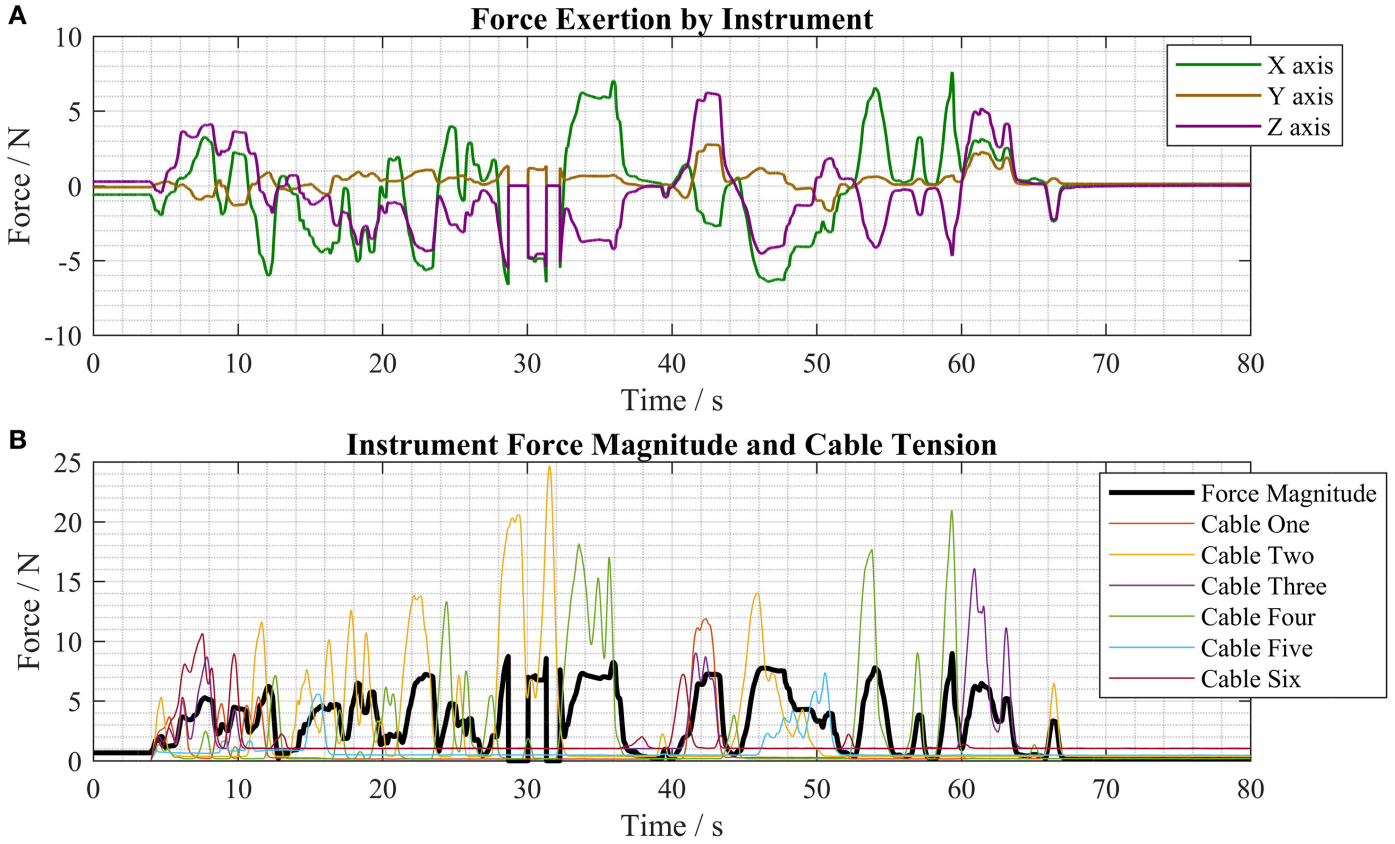
Force measurements taken during typical motions of the master controller **(A)** force exertion by the instrument in the X, Y, and Z axes **(B)** force magnitude and cable tension forces during the same test period.

The system was set up such that the tool, if it had been unconstrained, would be free to translate in the X, Y, and Z directions and rotate around the Y and Z axes. The measured forces in the X, Y, and Z axes are displayed in Figure 9A and the magnitude of the forces in the X, Y, and Z directions as well as the cable tensions in the force-transmission cables of the right instrument are displayed in Figure 9B. Full-bridge strain gauges (LCL-020, Omega Engineering, USA) were used to measure the tension in each of the cables, with readings sampled by a DAQ (Instrunet i100, GW Instruments, Inc., USA).

### Pre-clinical Validation

#### Collapsibility

**Figure 10:**
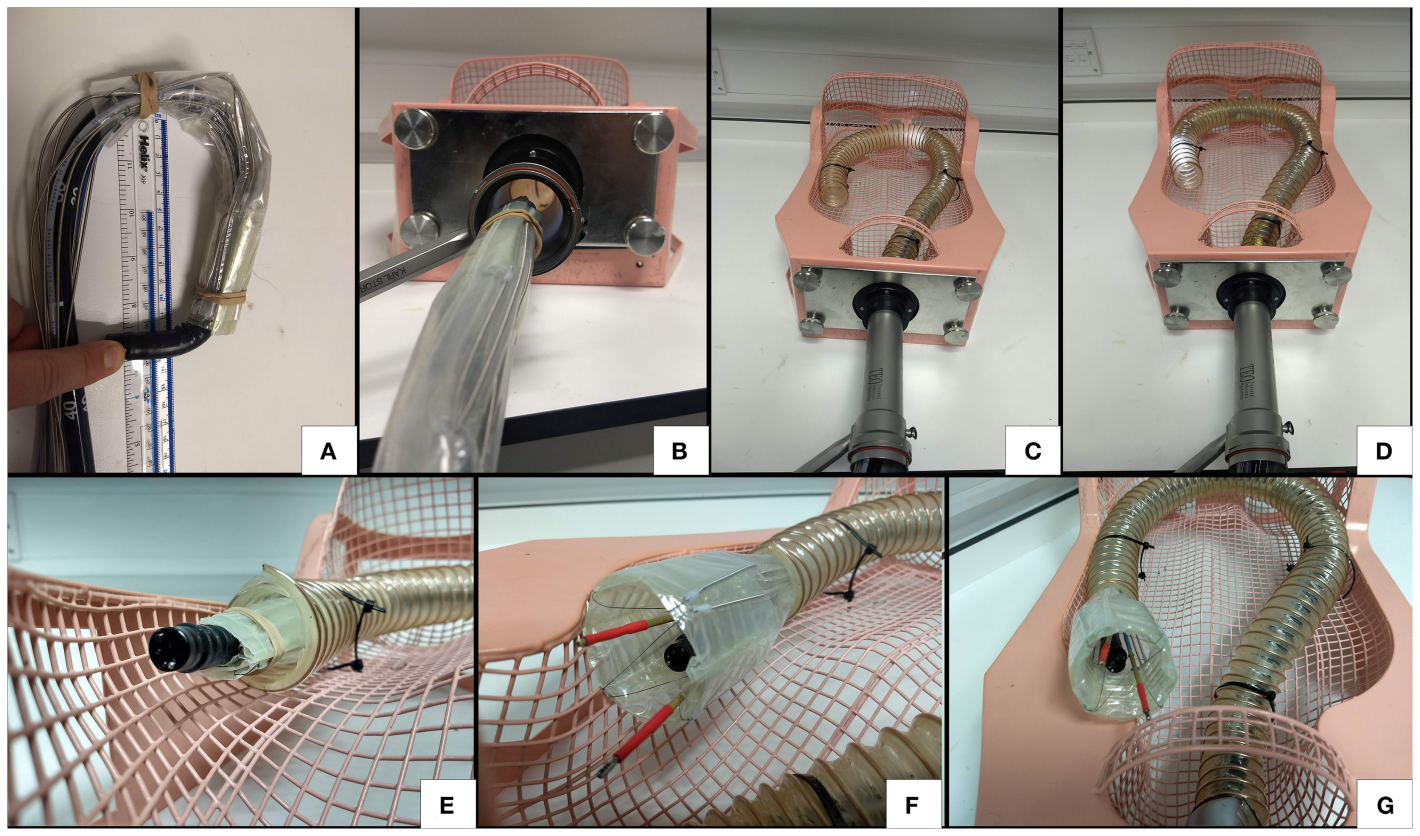
Demonstration of flexibility, insertion and deployment of the CYCLOPS **(A)** the endoscope is still free to bend **(B)** the CYCLOPS robot around the endoscope fits comfortably through a trans-anal port **(C)** the endoscope advances around curved, narrow path **(D)** further advancement **(E)** the assembly before deployment **(F)** deployed CYCLOPS robot ready to carry out surgical tasks **(G)** overview of robot after navigating the curved path and deploying.

The sheet material used in the construction of the inflatable structures has a thickness of 120 μm and this means that the structures can be folded into very low-profile shapes when the airtight chambers are empty. The thin PE/PET/PE material is flexible and therefore permits manipulation of the folded structure through tortuous paths. This functionality means that the deployable CYCLOPS robot can fold around an endoscope, deploy itself, perform a therapeutic or diagnostic function then be depressurised for retraction. Figure 10 shows the CYCLOPS assembly folded around a 12 mm flexible endoscope that maintains its ability to pass through a narrow, curved path, at the end of which the CYCLOPS robot is deployed.

The increase in volume after transition from the undeployed state to the deployed state was estimated by measuring the assembly in each state. The hexagonal prism structure was approximated as a regular hexagon with 28.5 mm long sides and 52 mm length, as in Figures 10F,G. With the undeployed CYCLOPS wrapped around a 12 mm endoscope, it was approximated as a cylinder 25 mm in diameter and 60 mm in length taking both the endoscope and structure around it as one entity, as in Figure 10E. Finally, the undeployed assembly without the endoscope was folded flat and approximated as a cuboid of cross-section 22 mm width by 11 mm height and having 60 mm length, see Figure 1A.

The percentage volume change was calculated using Equation (1), which yields the percentage difference in volume between an undeployed state and the deployed state.

(1) $$\begin{array}{r} \%\;Volume\;change\;=\;\left(\frac{V_{deployed}-V_{undeployed}}{V_{deployed}}\right)\times 100\% \end{array}$$

The respective volumes, ratios and volume change with respect to the deployed volume for these shapes are given in Table 2.

**Table 2:** Summary of volume change of deployable robot in various states.

**State**	**Deployed**	**Undeployed** **around scope**	**Undeployed folded**
Shape	Hexagonal prism	Cylinder	Cuboid
Dimensions	28.5 mm side, 52 mm length	25 mm diameter, 60 mm length	22 × 11, 60 mm length
Volume, V	109,734.9 mm^3^	29,452.4 mm^3^	14,520 mm^3^
Ratio V:V_deployed_	1:1	1:3.73	1:7.55
Volume change, %	–	73.16%	86.77%

#### Endoscopic Submucosal Dissection (ESD)

The grasper and diathermy instruments were inserted into the left and right CYCLOPS overtubes and the force-transmission cables coupled to the control system. Both chambers of the inflatable structure were pressurized at 2,000 mbar absolute using the syringe pump during the entire procedure. The haptic controllers move each instrument and toggle the grasper open or closed with available buttons, while a footswitch activates the diathermy instrument.

**Figure 11:**
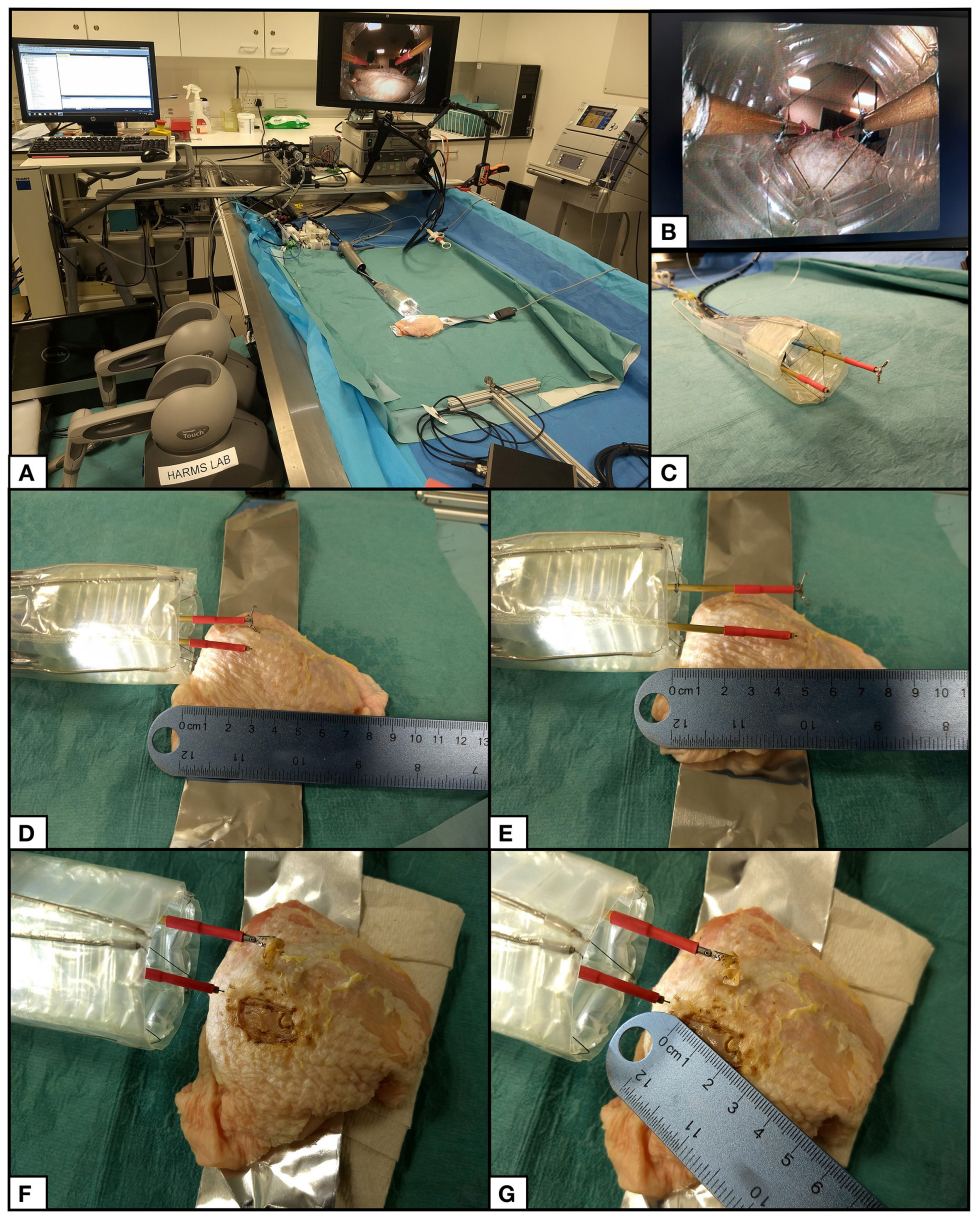
ESD procedure setup and results. **(A)** Setup of CYCLOPS system with haptic controllers, control computer, motor unit, syringe pump, imaging hub, diathermy unit, and grasper actuator. **(B)** View of inner structure from retracted flexible endoscope camera. **(C)** CYCLOPS robot in its calibration position. **(D)** Both instruments at minimum X position. **(E)** Both instruments at maximum X position. **(F)** Chicken breast after removal of skin. **(G)** Measurement of removed skin.

ESD requires three main steps: marking the lesion with the diathermy in a series of points encircling the cancer, then injecting saline solution into the submucosa to separate the target area from surrounding tissue and, finally, removing the cancerous tissue in one piece by making incisions following the marked points (Kume, 2009). The CYCLOPS robot was used to carry out these three steps on the skin of an *ex vivo* chicken breast sample to imitate this process, relying only on visual feedback from the endoscope, images of which are displayed in Figure 11. The endoscope was retracted to the rear of the CYCLOPS structure to show the hollow hexagonal prism shape from the interior, as shown in Figure 11B. The calibration position of the robot is shown in Figure 11C. Reach of the instruments of ~2 cm in the X direction is shown in Figures 11D,E. The area of skin removed was approximately equal to an ellipse with major and minor axes of 25 and 15 mm, displayed in Figures 11F,G.

## Discussion

Individual weld patterns have short completion times in the order of minutes, but this does not include preparation time. Preparation includes cutting sheet material from a roll, applying infrared absorbing dye and aligning individual layers. However, these additional steps might be handled in a process similar to that followed when using an inkjet printer to further reduce manufacture times. To use this laser to cut out welded designs, the appropriate laser power and scanning speed need to be identified. A small number of simple steps are all that is required to produce a ready to use soft robotic devices, which is a great advantage in comparison to conventional robotic systems with complicated assemblies.

This laser welding technique is currently limited to 2D weld patterns but 3D patterns could also be possible. However, a molding and demoulding method would be required, which is currently unnecessary with the simpler 2D system. The designs produced by this welding system can be scaled up further, only limited by the size of the robot and the vacuum table that clamps the material, and the welds created with this technique have a width of 1 mm, meaning that the designs can also be scaled down. Alternative optics could be used to focus the laser to a smaller spot size to reduce the track width and weld design with even finer details. For example, the shape-memory material Nitinol can be laser welded with a spot size of 0.4 mm for use in hermetically sealed medical devices (Khan et al., 2008).

In contrast to the laser welding/cutting method in Amiri Moghadam et al. (2018), this method does require a cutting step, in this case to manually remove excess plastic from around a completed part. However, there is no need for a heat pressing step that might damage the material or alter its properties. Furthermore, because the method described here does not both cut and weld the sheets simultaneously, soft robotic devices with multiple unique layers can be constructed and their designs can contain closed paths without causing the removal of material.

With equal pressure in both the circumferential and stiffening chambers, increasing the pressure increases the radial stiffness. This property can be exploited to prevent excessive force exertion on the colon and surrounding tissue. In applications that do not require large force exertion, for example if an optical fiber was placed in the CYCLOPS overtube and scanned over target tissue for diagnostic purposes, a lower stiffness support structure could be used. Diffuse Reflectance Spectroscopy (DRS) has been used to classify tissue in *ex-vivo* porcine colon (Avila-Rencoret et al., 2018) and multiple optical fibers can be integrated into a device to provide 360° scans of tubular organs (Avila-Rencoret et al., 2015). This would be a useful addition to the inflatable scaffold.

The overtubes are the only components that are not flexible or collapsible and a separate stiffening mechanism for the overtubes themselves would facilitate insertion of the device. However, any added bulk around the instruments will reduce the workspace, ultimately impacting the usability of the robot in this application that tackles gastric cancers of large size.

The programmatic design means that key design dimensions can be easily altered, including the width of the stiffening chambers. Changing the stiffening chamber width influences the stiffness, although further investigation is required to better understand how this occurs and how to strengthen its effect. The buckling of the 15 mm wide stiffening chambers may have affected their ability to resist collapse of the structure in comparison with the 12 mm wide stiffening chambers.

In contrast to the “pouch motors” in Niiyama et al. (2015), the inflatable chamber running along the corner of the structure is not coupled to rigid material but another inflatable chamber. This difference in construction leads to behavior that does not agree with the theory. As the stiffness in the stiffening beams exceeds the stiffness of the adjacent beams it is possible that the beams at lower pressure deform.

The laser welding manufacturing technique enables rapid manufacture of patient-specific or custom soft robotic devices, and the CYCLOPS robot can be customized by changing its geometry (Mylonas et al., 2014). For example, the workspace of each of the two instruments can be altered by changing where the cables connect to the instruments and by changing where the force transmission cables enter the support structure. The geometry of a laser welded CYCLOPS support structure and cable-routing sheath could be quickly and easily adapted based on patient data to ensure that the workspace of the robot is appropriate for the patient and the given surgical task. The inflatable structure can be disposed of after a single use because it is made from cheap thermoplastic film and is quick to produce. The instruments and force transmission cables can then be autoclaved in preparation for further use.

Long and rigid surgical instruments exert between 0.1 and 10 N when manually performing minimally invasive surgical tasks in the abdomen (Picod et al., 2005). Our results demonstrate that this deployable soft robotic device is capable of exerting forces comparable to those that are generated by manually operated laparoscopic instruments. Forces close to the maximum recorded forces in each axis, see Table 1, were regularly achieved during typical operation of the robot, as shown by Figure 9. Furthermore, the robot can still deliver high force exertion when attached to an endoscope that has navigated a long, curved path. The intuitive bimanual control of the CYCLOPS mechanism coupled with its high force exertion and large workspace make this a promising approach to robotically assisted MIS.

The instruments are controlled using two 6 DOF haptic controllers, which shows the system's potential for telemanipulation. Wearable devices could be linked with the system to provide tactile feedback and Virtual Reality (VR) headsets used to enhance this feedback (Maereg et al., 2017). The haptic controllers and visualization could be integrated together as in the DaVinci control system, although the footprint in an operating theater may be of concern. Cable driven manipulators have been used to measure contact force to enable high quality endomicroscopy images to be taken (Miyashita et al., 2018), so measured forces could be used as haptic feedback to the user. Being able to scale down the user's motion, control the needle used for injecting solution may reduce the number of people required to carry out ESD.

The measured cable tensions were similar to those measured when using rigid scaffold designs in previous work (Vrielink et al., 2018) but the force exertion of this system is significantly lower. This may be due to four factors: clamping of the CYCLOPS during testing, deformation of the inflatable structure, friction, and placement of the force sensor within the CYCLOPS workspace. The silicon sheath placed around the CYCLOPS during force measurements was seen to deform during testing, which lead to lower force transmission than if the structure had been held completely rigidly. However, clamping the inflatable structure with higher force would deform it, affecting the controllability and force exertion. Secondly, the deployable structure deforming under the loads of the tension forces applied around its boundaries is suspected to have caused losses in force exertion capability. Small errors in the real position and the actual position of the instruments caused by deformation of the support structure can cause lower tension in some cables, leading to lower exerted forces at the instrument tip. Friction of the cables within the PTFE tubing and Bowden cable may also have influenced force transmission, although to a lesser degree than the previous factors. Lastly, the forces exerted by each instrument depend on each instruments' position and orientation with respect to the local origin of the CYCLOPS. The placement of the force sensor in the CYCLOPS workspace therefore changes the maximum readings that can be achieved.

With the current setup, it is not possible to sense deformation of the inflatable structure during operation. With this capability, the robot's cable length based kinematic control scheme could compensate according to the deformation of the support structure to achieve more stable and accurate instrument movement. In addition, a control strategy based on the force exerted by the instruments is under development and aims to provide more robust control when deformation of the support structure is present. We are currently working on low-profile, multi-purpose soft sensors that can not only determine the shape of an inflatable chamber to solve this problem but also act as the actuation fluid (Avery et al., 2019). Shape sensing can be achieved through the use of optical fibers by measuring intensity modulation (Sklar et al., 2016) or wavelength and frequency modulation (Wang and Liu, 2018). Problems with these approaches include a lack of information on bending direction or locality and elevated costs. We did not consider sensing using optical fibers for these reasons.

Other examples of future work include the integration of sensors for proprioception, exteroception, and to collect diagnostic information from the patient. A more compliant sheath with anchoring capabilities would prevent unwanted movement of the CYCLOPS when grasping and exerting force on its surroundings.

## Data Availability Statement

The datasets generated for this study are available on request to the corresponding author.

## Author Contributions

MR designed, manufactured, and tested the soft robotic devices. MZ and MR set up the system. AD and GM supervised the research. MR, JA, and GM worked on the manuscript.

### Conflict of Interest

The authors declare that the research was conducted in the absence of any commercial or financial relationships that could be construed as a potential conflict of interest.
